# Pickering-Stabilized Breath Figure Assembly of CNWs-TiO_2_/PLA Porous Films: Synergistic Reinforcement and Functional Grading

**DOI:** 10.3390/polym18131602

**Published:** 2026-06-28

**Authors:** Ting Zhang, Junhao Liang, Bin Wang, Bohua Wen, Zhengyang Xi, Xuyang Zhao, Xinhai He

**Affiliations:** 1 School of Materials Science & Engineering, Xi’an Polytechnic University, Xi’an 710048, China; liangjunhao@xpu.edu.cn (J.L.); wangbin_1120@163.com (B.W.); 18335471077@163.com (B.W.); x18792912638@163.com (Z.X.); wy3069343052@163.com (X.Z.); 2 Xi’an Key Laboratory of Textile Composites, Xi’an Polytechnic University, Xi’an 710048, China; 3 Engineering Research Center of Composites Weaving and Functional Technology, Universities of Shaanxi Province, Xi’an 710048, China

**Keywords:** porous PLA film, polylactic acid, cellulose nanocrystal whiskers, nano titanium dioxide, breath figure method

## Abstract

(1) Background: The development of biodegradable polymers with enhanced functionality is critical for advancing sustainable packaging technologies. This study addresses the challenge of simultaneously improving the structural rigidity and functional performance of poly(lactic acid) (PLA) materials. (2) Methods: Cellulose nanowhisker (CNW) and TiO_2_ nanoparticle (Nano-TiO_2_) reinforced PLA porous composite films were fabricated via a nanoparticle-assisted breath figure method. The effects of these hybrid nanoparticles on the morphology, thermal stability, and structure of the resulting composites were systematically investigated. (3) Results: The incorporation of CNWs and Nano-TiO_2_ played a dual role: they acted as Pickering-like stabilizers at the water/polymer interface, preventing droplet coalescence and facilitating the formation of a well-defined honeycomb-like porous structure. They also significantly enhanced the relative crystallinity of the PLA matrix from 20.26% to 36.31%, owing to the heterogeneous nucleation effect. Consequently, the mechanical properties were significantly enhanced, with the maximum tensile strength and Young’s modulus increasing by 123.3% and 21.9%, respectively. Furthermore, the composite films exhibited excellent UV-shielding performance, achieving an SR UV-B of 97.6%. (4) Conclusions: The synergistic reinforcement of CNWs and Nano-TiO_2_ effectively endows PLA composites with superior mechanical properties and functional protection. These findings establish the CNWs-Nano-TiO_2_/PLA composite film as a promising candidate for high-performance smart packaging applications.

## 1. Introduction

Polymer porous films become a center of interest thanks to their extremely high specific surface areas and large variety of applications, such as biotechnology, catalysis, sensors, photovoltaics, templates, etc. [1,2,3,4,5]. Among various fabrication techniques, the breath figure (BF) method [6,7,8] stands out as a simple and effective approach to forming pore structures on the polymer surface in one step due to its merits of being low in cost, saving time, and being easy to operate [9,10,11,12]. Typically, the breath figure method involves a hydrophobic polymer dissolved in a volatile solvent that is immiscible with water. During the casting of a polymer solution in a humid environment (normally with relative humidity above 50%), evaporative cooling of the solvent can cause water molecules from the surrounding air to condense on the polymer film surface [11,13,14].

Poly (lactic acid) (PLA) porous films have broad applications in the field of tissue engineering scaffolds and drug-controlled release systems because of their excellent biodegradability and biocompatibility [15]. However, the brittleness, poor toughness, low melt strength and thermolability, and lack of functional properties of PLA restrict its widespread application in high-end packaging [1]. Conventional strategies to address these limitations have primarily involved physical blending with a plasticizer [12], nucleating agent, or inorganic reinforcements [16,17]. While the individual reinforcement of PLA with CNWs or TiO_2_ has been extensively documented to improve mechanical and functional profiles, achieving a stable and uniform porous architecture remains a formidable challenge.

CNWs possess abundant sources, good biocompatibility and biodegradability, renewability, excellent thermal stability, cost-effectiveness, non-toxicity, and a large and tunable surface area [16,18,19,20,21]. Lau Kia Kian et al. [21,22] fabricated a new dual-layer membrane composed of PLA and poly (butylene succinate) (PBS) with CNWs as a functional filler. The results showed that membranes with 3% CNWs displayed a better tensile strength, elongation at break, and Young’s modulus compared to neat samples. E.F. Sucinda [16] developed films by using Napier cellulose nanowhisker-reinforced PLA, and the PLA/NWC films showed good dispersion, high crystallinity, high tensile strength, and a high tensile modulus at NWC concentrations of 1.0 wt% and 1.5 wt%. This is attributed to the excellent dispersion of NWCs in the PLA matrix, which has the highest crystallinity. Kuichuan Sheng [23] prepared a PLA/bamboo cellulose nanowhisker (BCNW)/silane surface-modified ultrafine bamboo-char (UFBC) bionanocomposite with a higher tensile strength (18.87 MPa) and tensile modulus (272.24 MPa) because UFBC dispersed uniformly in the PLA matrix, and the two phases had a good interface effect.

Nano-TiO_2_ is relatively inexpensive, non-toxic, highly resistant to various chemicals, and has a high potential for UV absorption [24]. Several research studies have been carried out to understand and explore the effects of TiO_2_ on improving PLA properties. A. Shebi et al. [25] fabricated hierarchically porous PLA-TiO_2_ nanocomposite films, which have shown tremendous improvement in antibacterial activity against *S. aureus* under visible light or in darkness. Linyu Shao et al. [26] developed an innovative composite film by integrating PLA, PCL, and TiO_2_ nanoparticles through physical blending. The composite films exhibit favorable mechanical properties. The UPF value of the PLA/PCL/TiO_2_ composite film is approximately 201, about 40 times higher than that of the TiO_2_-free composite film, indicating remarkable UV resistance. Jayanta Kumar Nayak et al. [27] synthesized TiO_2_-strengthened PLA nanocomposites and found that the nanocomposite had specific antibacterial properties. Neda Tajari [28] also found that the addition of TiO_2_ nanoparticles can introduce antimicrobial properties to PLA film. Pattamaphon Chanklom [29] incorporated TiO_2_-anatase into PLA for use as a photocatalyst using the blown film method. It was found that the PLA/TiO_2_ composite film was a highly promising photocatalyst for the degradation of benzene.

Although PLA/CNW and PLA/TiO_2_ composites have been widely reported, most previous studies treat these fillers as passive reinforcements incorporated through melt-blending or solution-casting, where agglomeration and limited interfacial interaction remain persistent challenges. Unlike these conventional approaches, we propose a dual-functional Pickering stabilization strategy based on the breath figure (BF) mechanism to fabricate functionalized porous PLA films in this study. By incorporating cellulose CNWs and Nano-TiO_2_ as Pickering-like stabilizers, we achieved precise control over the morphology of the porous structure. CNWs serve simultaneously as mechanical reinforcements and emulsion stabilizers, while Nano-TiO_2_ acts as both a UV-shielding agent and an interfacial co-stabilizer. This strategy not only suppresses filler aggregation during film formation but also promotes the in situ assembly of a hybrid CNW-TiO_2_ network within the PLA matrix. As a result, the composite achieves a synergistic balance of enhanced stiffness, improved crystallinity, and excellent UV-shielding, which is difficult to realize using traditional processing routes. This dual-enhancement strategy, addressing both hierarchical structure and intrinsic performance, offers a simple and efficient approach to developing high-performance PLA-based functional porous films.

## 2. Materials and Methods

Poly (L-lactic acid, 4032D) with an average molecular weight of 10 × 10^4^ was purchased from Natureworks LLC. Minnetonka, MN 55345, USA. Carboxylate cellulose nanowhiskers (CNWs) were purchased from Guilin Qi Hong Technology Co., Ltd., Guilin, China, and the CNWs were nanorods with a width of 4–10 nm and a length of 100 to 500 nm. Nano-TiO_2_ (Nano-TiO_2_) was purchased from Shanghai Aladdin Biochemical Technology Co., Ltd. Shanghai, China. All the materials were used as received, and all the solvents were also used as received, without any further purification, including dichloromethane (CH_2_Cl_2_ or DCM, 99.0%, Tianjin Damao Chemical Reagent Factory, Tianjin, China) and anhydrous ethyl alcohol (C_2_H_5_OH, AR, Tianjin Fuyu Fine Chemical Co., Ltd., Tianjin, China). Deionized water was self-made in the laboratory.

**Figure 1:**
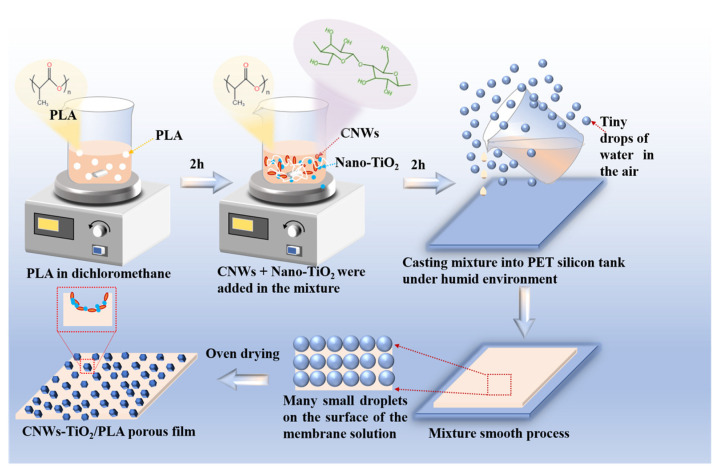
Schematic of the preparation process of the CNWs-Nano-TiO_2_/PLA porous film.

Preparation of CNWs-Nano-TiO_2_/PLA porous film: CNWs-Nano-TiO_2_/PLA porous film was prepared through a simple solution-casting method combined with a breath figure method without the need for any chemical modification or additives. Preliminary experiments were conducted to optimize the CNW and TiO_2_ contents. Loadings ranging from 0.1 to 1.0 wt% CNWs and 0.1 to 1.0 wt% TiO_2_ were evaluated. The 1 wt% CNW/0.5 wt% TiO_2_ formulation was selected as the optimal composition, offering the best balance of dispersion, mechanical properties, and UV-shielding performance. The preparation process is shown in Figure 1. Step 1: A total of 5 g of PLA particles was dissolved in 100 g of dichloromethane and stirred at 300 rpm for 2 h to form a homogeneous solution for future use. Step 2: A 30.0 g PLA solution (5 wt%) was weighed in a beaker, and then 0.3 g of CNWs and 0.15 g of Nano-TiO_2_ powder were added into the beaker and stirred at 300 rpm for 2 h. During this process, the solution transformed from transparent to a white emulsion. Step 3: The prepared mixture was cast into a PET silicon membrane tank (length: 70 mm, width: 50 mm) uniformly and moved into a bake out furnace with a humidity of about ~70% at 60 °C for 6 h to get 1% CNWs-0.5% Nano-TiO_2_/PLA porous films.

The morphology and microstructure of the samples were examined with a field emission scanning electron microscopy (FESEM; QUANTA-450-FEG, TEXTEST AG, Schwerzenbach, Switzerland). A Fourier transform infrared spectrometer (FT-IR, Nicolet is50, Thermo Fisher, Waltham, MA, USA) was used to detect the functional groups in the CNWs-Nano-TiO_2_/PLA composite film. X-ray diffraction (XRD; DX-2700BH, Dandong, China) with Cu Kα1 radiation (l = 1.5418 A) was used for phase identification. Thermogravimetric Analysis (TGA, Q500, TA Instruments, New Castle, DE, USA) was used to evaluate the thermal stability of the samples. The UV–visible diffuse absorption spectrum was measured using a spectrophotometer (UV, UV-2600, SHIMADZU, Kyoto, Japan) with a measurement range of 200–800 nm. The surface roughness of the PLA films was examined using an atomic force microscope (AFM, CSPM 5500A, Beijing, China). The water contact angle of the film surface was measured using a contact angle meter (JGW-360A, Chengde, China) to evaluate the change in hydrophilicity of the film surface. The measurement temperature was room temperature; the substance of the surrounding phase was distilled water. The final measurement results were taken as the average of three measurements. The 3D topology of the composite film was characterized using a confocal laser scanning microscope (CLSM, OLS5100-SAF, Olympus Corporation, Tokyo, Japan).

## 3. Results and Discussion

### 3.1. Structure Characterizations

**Figure 2:**
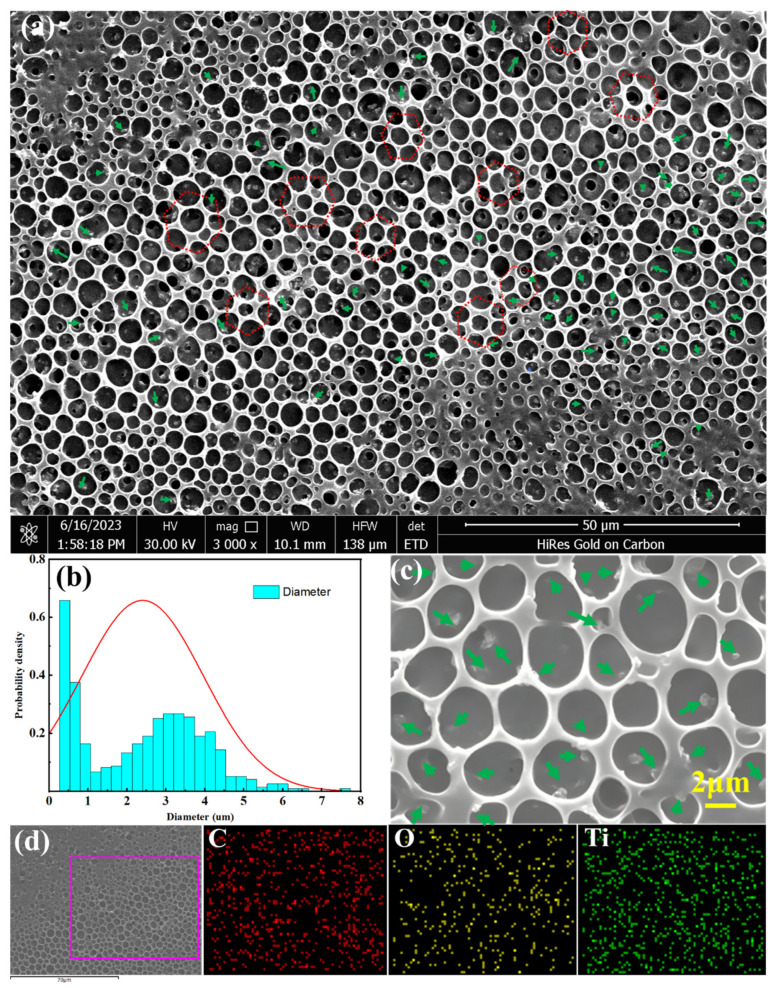
SEM and EDS images of microscopic structure of (**a**) morphology of 1%CNWs-0.5%Nano-TiO_2_/PLA composite film; (**b**) SEM image of 1%CNWs-0.5%Nano-TiO_2_/PLA composite film with greater magnification; (**c**) pore size distribution map generated using ImageJ software (ImageJ 1.54g, National Institutes of Health, USA) based on (**a**); (**d**) EDS mapping of 1%CNWs-0.5%Nano-TiO_2_/PLA composite film.

Figure 2 shows the surface microstructure of the 1%CNWs-0.5%Nano-TiO_2_/PLA composite film. Figure 2a displays a low-magnification SEM image of the CNWs-Nano-TiO_2_/PLA film. As one can see in Figure 2a, hexagonally ordered breath figure arrays were formed. The surface of the film has many pores; one pore is surrounded by six other pores (red dashed hexagon), which reflects the shape of the water droplet template. The green arrows indicate CNWs or Nano-TiO_2_. It can be seen that the walls of each pore have CNWs or Nano-TiO_2_ (indicated by green arrows). However, although porous films with ordered structures could have been formed, dramatic differences in the pore diameters are found in Figure 2a, which are caused by the introduced CNWs and Nano-TiO_2_. The chemical structures, topology, and molar mass of film-forming materials have significant influences on the morphologies of BF porous films, as has been reported recently [30]. To evaluate the statistical homogeneity of the porous structure of the composite film, the pore size distribution was analyzed based on 904 randomly selected pores from Figure 2b using Image J; the results are shown in Figure 2b. As shown in Figure 2b, the composite film exhibits a unimodal distribution, with an average pore diameter of 2.49 ± 1.36 μm. Figure 2c displays a high-magnification image of the CNWs-Nano-TiO_2_/PLA film. It is noteworthy that each pore is embedded with TiO_2_ nanoparticles or CNWs, and some of them are indicated by a green arrow. CNWs and TiO_2_ nanoparticles both migrated to the water/dichloromethane interface, acting as Pickering stabilizers to prevent droplet coalescence and suppress the disordered convection, thereby preserving the integrity of the porous structure. The adsorption of hydrophilic CNWs and TiO_2_ nanoparticles at the water/solvent interface effectively lowers the interfacial tension, thermodynamically favoring the stabilization of water droplets during the breath figure process. The uniform pore size and regular arrangement of the honeycomb structure suggest that the CNWs and TiO_2_ provided effective interfacial stabilization, suppressing droplet coalescence during solvent evaporation. Although direct in situ visualization was not performed, the final morphology aligns well with classical Pickering–breath figure systems reported in the literature [12], supporting the proposed mechanism. Figure 2d is the EDS mapping of the CNWs-Nano-TiO_2_/PLA film. The pink-purple box in Figure 2d indicates the selected area for EDS analysis. As one can see from Figure 2d, there are C, O, and Ti elements in the films. Among them, C and O may come from both the PLA and CNWs, while Ti comes from Nano-TiO_2_.

**Figure 3:**
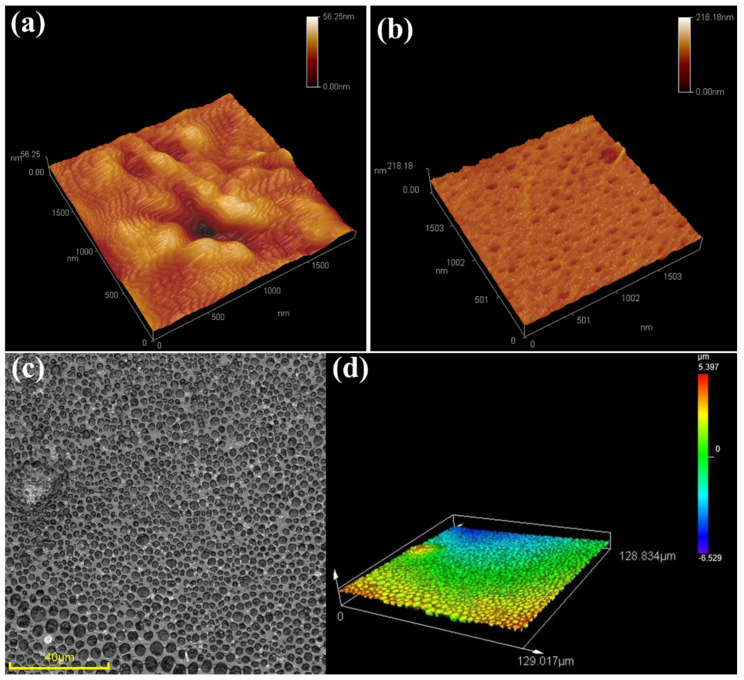
Surface microscopic structure of CNWs-Nano-TiO_2_/PLA composite film: (**a**) AFM images of 5%PLA film; (**b**) AFM images of 1%CNWs-0.5%Nano-TiO_2_/PLA composite film; (**c**) 2D CLSM images of a CNWs-Nano-TiO_2_/PLA composite film. (**d**) 3D CLSM images of a CNWs-Nano-TiO_2_/PLA composite film.

Figure 3a,b show AFM images of 5%PLA film and 1%CNWs-0.5%Nano-TiO_2_/PLA composite film, respectively. As one can see in Figure 3a,b, the neat PLA film displays a relatively smooth surface, which is characteristic of the polymer matrix’s intrinsic topography. In contrast, the composite film exhibits a significantly rougher surface. The incorporation of CNWs and TiO_2_ nanoparticles dramatically increased the surface roughness from ~56 nm (neat PLA film) to ~218 nm (1%CNWs-0.5%Nano-TiO_2_/PLA composite film). This emergence of nanoscale asperities can be attributed to the embedded nanoparticles and the phase heterogeneity at the polymer–filler interface. Furthermore, Figure 3b shows micropores induced by the breath figure method. The nanoscale roughness and micropores establish a hierarchical texture, which is critical for enhancing wettability properties.

Figure 3c,d present confocal laser scanning microscopy (CLSM) images of the obtained CNWs-Nano-TiO_2_/PLA composite film. It can be seen from Figure 3c that a highly ordered, honeycomb-like porous pattern was successfully fabricated, which is consistent with the SEM observations. The pores are densely packed with a relatively uniform diameter distribution. The formation of this regular array suggests that the CNWs and TiO_2_ nanoparticles played a pivotal role in stabilizing the water droplets during the solvent evaporation process. The 3D height map of Figure 3d displays good macroscopic planarity across the scanned area (129 × 129 μm), indicating that the self-assembly process induced by the breath figure method yields a homogeneous structure not only locally but also over larger domains. The color gradient (from blue valleys to red peaks) clearly delineates the depth of the honeycomb cavities. The 1%CNWs-0.5%Nano-TiO_2_/PLA composite film exhibits a high arithmetical mean height (Sa) of 0.714 μm and a root mean square height (Sq) of 0.934 μm. This substantial micro-roughness confirms that the introduction of CNWs and TiO_2_ nanoparticles, coupled with the breath figure process, can successfully be used to construct a hierarchical micro/nano-textured surface.

**Figure 4:**
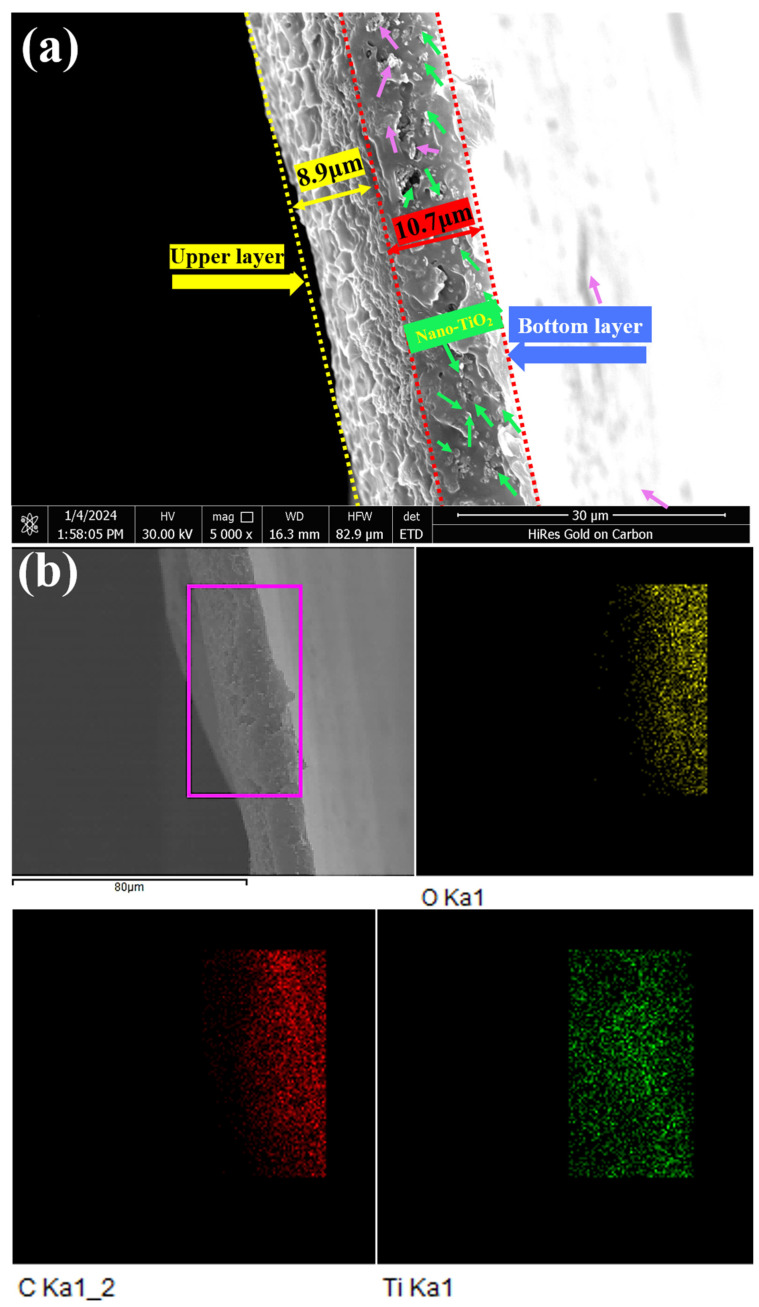
Cross-section SEM image of CNWs-Nano-TiO_2_/PLA composite film: (**a**) low magnification; (**b**) EDS mapping of cross-section of CNWs-Nano-TiO_2_/PLA composite film.

Figure 4 shows the cross-section microstructure of the CNWs-Nano-TiO_2_/PLA composite film. Interestingly, it can be seen that the composite film has a distinct bilayer structure with nanoparticle sedimentation in the bottom layer: namely, an upper layer (about 8.9 μm thick, marked by a yellow broken line) and a bottom layer (about 10.7 μm thick, marked by a red broken line) that is densely populated with inorganic aggregates (indicated by green and purple arrows). The cross-sectional SEM image also reveals that the composite film possesses an asymmetric, gradient structure along the vertical direction. The uppermost layer, which constitutes the primary porous region and exhibits a rough and uneven morphology, localizes Nano-TiO_2_ particles and CNWs within the pore walls, thereby rendering them invisible on the cross-sectional surface. Meanwhile, the bottom layer has many visible Nano-TiO_2_ particles marked with green arrows uniformly distributed at the bottom layer, and CNWs marked with purple arrows are partially aggregated. This vertical heterogeneity is attributed to the gravitational sedimentation effect. During the solvent evaporation process, the significantly higher density of Nano-TiO_2_ nanoparticles (3.9 g/cm^3^) and CNWs (1.6 g/cm^3^) compared to the PLA matrix (1.24 g/cm^3^) and solvent (1.33 g/cm^3^) drives them to settle towards the substrate side before the solution viscosity increases sufficiently to immobilize them. This spontaneous stratification creates a functionally graded material. The polymer-rich upper layer facilitates the formation of ordered breath figure arrays (as observed in the surface SEM) without severe disruption from large inorganic aggregates, while the inorganic-rich bottom layer may potentially enhance the mechanical integrity or functional performance (photocatalytic activity) of the composite film. Additionally, the CNWs likely serve as a physical barrier to prevent the severe agglomeration of Nano-TiO_2_. The coexistence of CNWs and Nano-TiO_2_ creates a hierarchical microstructure, potentially offering synergistic functional properties such as mechanical reinforcement, UV-shielding, and antibacterial activity, without compromising the film’s structural cohesiveness.

Figure 4b is the EDS mapping of the CNWs-Nano-TiO_2_/PLA film cross-section and shows that there are C, O, and Ti elements in the films. The carbon signal (red) is attributed to the organic nature of both the polymer matrix and the CNW reinforcement. The oxygen signal (yellow) reflects the oxygenated functional groups in the matrix, which originate from the cumulative oxygen content of the TiO_2_ and CNW fillers. The titanium element (green) is distributed throughout the entire thickness, which corresponds to the Nano-TiO_2_ particles. Cross-sectional EDS mapping revealed a gradual decrease in Ti (from TiO_2_), O, and C (associated with CNWs) from the top to the bottom of the film, confirming a functionally graded nanoparticle distribution. The formation of the bilayer structure is driven by the density mismatch between the nanofillers (ρ_TiO_2__ ≈ 3.893 g/cm^3^, ρ_CNWs_ ≈ 1.6 g/cm^3^) and the evaporating PLA/solvent medium. Heavier TiO_2_ particles settle preferentially, accumulating near the substrate, while lighter CNWs remain partially entrained in the upper region, resulting in a vertically graded composite architecture.

### 3.2. Structure–Property Relationship of Films

**Figure 5:**
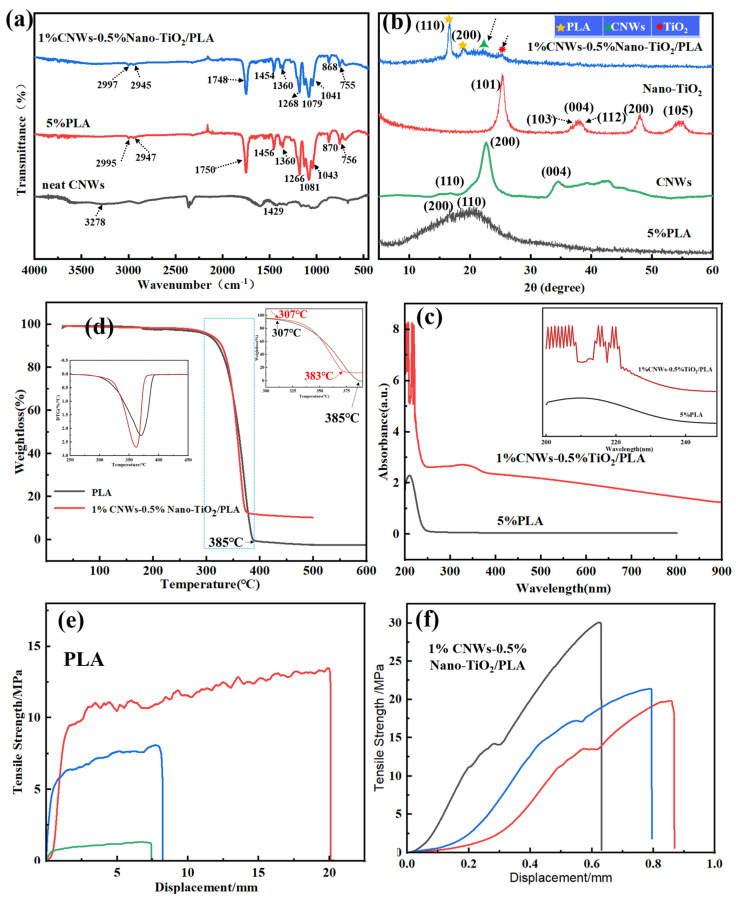
FT-IR, XRD, UV-vis, TGA and tensile strength results of the films: (**a**) FT-IR results; (**b**) XRD results; (**c**) UV-vis curve; (**d**) weight loss curve and DTA curve; (**e**) tensile strength results of neat PLA film; (**f**) tensile strength results of 1%CNWs-0.5%Nano-TiO_2_/PLA composite film.

FT-IR spectra, XRD, UV-vis spectra, TGA, and the tensile strength results of neat PLA film and 1%CNWs-0.5%Nano-TiO_2_/PLA composite film are respectively depicted in Figure 5. As one can see from Figure 5a, the conventional bands for CNWs were noted at 3278 cm^−1^ (O-H stretching), 2900 cm^−1^ (C-H stretching vibration), 1429 cm^−1^ (symmetric CH_2_ bending), and 1329 cm^−1^ (CH_2_ wagging at C-6). The peaks at 1166 cm^−1^, 1118 cm^−1^ and 1059 cm^−1^ correspond to asymmetric stretching vibration peaks of the C-O-C bonds in CNWs [31]. The peaks of neat PLA film at 2995 cm^−1^ and 2947 cm^−1^ were associated with the C-H asymmetric and symmetric stretching vibrations of aliphatic -CH_3_ groups, respectively. The vibrational peak at 1750 cm^−1^ is attributed to the stretching vibration of the carbonyl groups, C-O, of the ester group. The peaks at 1456 cm^−1^ and 1385 cm^−1^ represent asymmetric and symmetric bending of the C–H bond in the methyl groups, while the peaks at 1266 cm^−1^ and 1043 cm^−1^ are related to the C-O-C symmetric and asymmetric stretching [32,33]. With the addition of CNWs and Nano-TiO_2_, no new characteristic peaks or obvious shifts in peaks were observed. This indicated that no new covalent bonds were formed after the incorporation of modified CNWs and TiO_2_ [32,34]. Instead, the interfacial coupling is entirely governed by physical interactions, specifically intermolecular hydrogen-bonding and interfacial physical adhesion. The dense hydroxyl groups (-OH, 3278 cm^−1^) acting as hydrogen donors on CNW surfaces engage in localized physical interactions with the electronegative carbonyl oxygen atoms (-C=O, 1750 cm^−1^) and ether linkages (C-O-C) of the PLA chains. This induces subtle variations in peak intensity and local electronic cloud distribution without altering the primary covalent backbone. From a micromechanical perspective, this extensive physical hydrogen-bonding network, combined with the structural confinement of Nano-TiO_2_, provides a strong thermodynamic driving force that suppresses nanofiller agglomeration and promotes interfacial adhesion. Under external mechanical loading, these dynamic, reversible physical bonds function as “sacrificial bonds” that effectively transfer stress and dissipate mechanical energy, thereby contributing significantly to the macro-performance enhancements observed in the composite film.

Crystallinity analysis was conducted to evaluate the impact of CNWs and Nano-TiO_2_ on the crystallinity of composite films. Figure 5b shows the XRD pattern of the samples. For PLA film, it can be seen that the crystallinity of pure PLA film is poor, mainly characterized by a broad peak that is assigned to the 2θ = 16.7° (200) and 22.3° (110) planes. The XRD diffraction pattern for CNWs exhibited peaks at 2θ = 16.7, 22.5 and 34.6 at (1 1 0), (2 0 0) and (0 0 4) planes, respectively, which correspond to the characteristic peaks of cellulose structure [35,36]. For the Nano-TiO_2_ sample, XRD studies showed that the TiO_2_ nanoparticles are anatase, and all the peaks at 2θ = 25.3°, 36.9°, 37.8°, 38.6°, 48.0°, 53.9° and 55.0 correspond, respectively, to the planes (1 0 1), (1 0 3), (0 0 4), (1 1 2), (2 0 0), (1 0 5) and (2 1 1), which matches well with the standard diffraction peaks of anatase TiO_2_ (JCPDS card No. #21-1272). For the 1%CNWs-0.5%Nano-TiO_2_/PLA composite film, a sharp peak at around 16.7° (plane 110) and a small sharp peak at 19.0° (plane 200) can clearly be seen. Meanwhile, very faint peaks were observed for the 1%CNWs-0.5%Nano-TiO_2_/PLA composite film at 22.5° and 25.3°, indicating cellulose and TiO_2_ crystallinity, respectively. By comparison, we also found that the peak intensity at 16.7° and 19.0° for the 1%CNWs-0.5%Nano-TiO_2_/PLA composite film was higher than that of the pure PLA film. This can be explained by the presence of CNWs and TiO_2_ in the PLA composite film. The excellent crystallinity of CNWs and Nano-TiO_2_ naturally increased the crystallinity of the 1%CNWs-0.5%Nano-TiO_2_/PLA composite film. Meanwhile, CNWs and TiO_2_ serve as nucleating agents, leading to many crystallization nucleation points, which accelerated crystallization and led to the higher crystallinity of the 1%CNWs-0.5%Nano-TiO_2_/PLA composite film [37]. However, the characteristic peak intensity of the CNWs and Nano-TiO_2_ is significantly reduced due to the nanoparticles being encapsulated within the PLA matrix.

The optical properties of the PLA and 1%CNWs-0.5%Nano-TiO_2_/PLA composite film were determined using a UV–Visible Spectrophotometer. Figure 5c shows the UV–Vis absorbance spectra of the films, and Table 1 shows the quantitative optical properties of the PLA-based films. By combining the two sets of data, we can conclude that the 1%CNWs-0.5%Nano-TiO_2_/PLA composite film has a relatively high UV absorption and still maintains relatively high transparency (71.4%) in the visible range. Neat PLA exhibits an extremely low absorbance (0.05) and UV-B shielding rate (SR*_UV-B_* = 10.9%), indicating poor resistance to UV-B radiation. In contrast, the 1%CNWs-0.5%Nano-TiO_2_/PLA composite film shows a dramatic increase in absorbance (1.62) and SR*_UV-B_* (97.6%). This enhancement arises from the synergistic effect of CNWs and Nano-TiO_2_. Nano-TiO_2_ is a wide-bandgap semiconductor and strongly absorbs UV radiation via electronic transitions [29]. For neat PLA, the absorbance at 360 nm is merely 0.02, with an SR*_UV-A_* of 4.5%. The absorbance of the 1%CNWs-0.5%Nano-TiO_2_/PLA composite film rises to 0.88, and SR*_UV-A_* reaches 86.8%. The high UV-A shielding efficiency confirms that the composite film can effectively block longer-wavelength UV radiation, which is critical for protecting materials from photoaging.

The composite film exhibits excellent UV-blocking efficiency while retaining moderate visible transparency. This balance is primarily attributed to the judicious selection of Nano-TiO_2_ loading and its homogeneous dispersion within the PLA matrix. At 0.5 wt%, TiO_2_ particles effectively absorb and scatter UV photons without introducing excessive scattering in the visible spectrum. Meanwhile, the CNWs form a nanoscale network that does not significantly disrupt light transmission. As a result, the film achieves a visible light transmittance of 71.4% at 550 nm, which is sufficient for most rigid packaging and labeling applications. Although not optically perfect, the combination of high UV-shielding, adequate clarity, and fully bio-based composition supports its potential for short-term packaging applications where product visibility and UV protection are simultaneously required.

Figure 5d presents the weight loss curve of neat PLA film and 1%CNWs-0.5%Nano-TiO_2_/PLA. The TG curve analysis shows that the investigated temperature ranged from room temperature to 600 °C. It can be seen that the weight losses of PLA and the composite film are mainly in the range of 300–385 °C. PLA and its composite films all began to decompose after ~200 °C, and the curves overlap together. The max degradation rate for neat PLA film begins at 289 °C and ends at 383.3 °C. The max degradation rate for 1%CNWs-0.5%Nano-TiO_2_/PLA composite film begins at 300 °C and ends at 383 °C. The addition of both TiO_2_ nanoparticles and CNWs makes the initial and final degradation temperatures of the CNWs-Nano-TiO_2_/PLA composite film similar to those of neat PLA film. However, the difference is that the neat PLA film has a 100% thermal weight loss, while the CNWs-Nano-TiO_2_/PLA composite film has an 88% thermal weight loss. Figure 5e shows the DTG curves of the films. The DTG data confirm that this CNWs-Nano-TiO_2_/PLA composite film undergoes faster thermal decomposition (deeper peak) than pure PLA; this may be attributed to a synergistic effect. Although the onset degradation temperatures of neat PLA and the composite film are similar, the composite exhibits a significantly faster decomposition rate, as evidenced by the sharper DTG peak. This behavior can be attributed to the combined influence of porosity and filler–matrix interactions. First, the porous architecture generated by the breath figure process provides interconnected channels that facilitate the outward diffusion of volatile degradation products, thereby reducing mass transfer resistance. Second, the extensive CNW–TiO_2_/PLA interfaces create a large number of imperfect crystalline regions and defect sites, which act as preferential initiation points for thermal scission. As a result, once degradation is triggered, the composite film undergoes more rapid chain breakdown despite having a higher overall crystallinity.

In addition, although XRD results indicate an increase in the overall crystallinity of the 1 wt% CNWs/0.5 wt% Nano-TiO_2_/PLA composite film, this does not necessarily imply a uniform enhancement of chain ordering throughout the entire matrix. The improved diffraction intensity primarily arises from the heterogeneous nucleation effect of CNWs and Nano-TiO_2_, which promotes localized crystallization and facilitates the formation of more numerous but smaller crystallites. These fine crystals, although increasing the total crystalline fraction, also introduce a large number of imperfect crystalline regions and crystal–amorphous interfaces. The pore structure of CNWs-Nano-TiO_2_/PLA composite film creates additional interfaces between the PLA matrix and the fillers (CNWs, Nano-TiO_2_). At these interfaces, polymer chains tend to be partially constrained yet remain less perfectly packed compared with bulk crystals. Consequently, while the global crystallinity increases, the average perfection and thermal stability of the crystalline regions may be compromised. This explains why the composite exhibits a lower onset degradation temperature and a sharper DTG peak despite its higher crystallinity. Furthermore, the porous or loosely packed structure around the fillers provides additional diffusion pathways for volatile degradation products, reducing mass transfer resistance and accelerating the decomposition process. The residual char (~12 wt%) after 400 °C further confirms that the fillers themselves do not degrade but act as physical barriers that alter the degradation pathway rather than preventing thermal decomposition. Therefore, the seemingly contradictory trends between XRD and TG results can be rationalized by considering crystallite size distribution, crystal perfection, and interfacial effects, rather than crystallinity alone.

The tensile strengths of neat PLA film and CNWs-Nano-TiO_2_/PLA composite film were measured, and the representative stress–displacement curves are shown in Figure 5e,f. The mechanical properties are summarized in Table 2. The tensile strength of the 1%CNWs-0.5%Nano-TiO_2_/PLA composite film has been significantly improved: while PLA exhibited a maximum tensile strength of 13.48 MPa, the 1%CNWs-0.5%Nano-TiO_2_/PLA composite film reached 30.1 MPa, which is an increase of 123.3%. The incorporation of CNWs and Nano-TiO_2_ significantly improved the stiffness of the PLA-based composite film. As shown in Figure 5f, the Young’s modulus increased from 0.32 GPa (neat PLA) to 0.39 GPa (composite film), representing an enhancement of approximately 21.9%. This significant reinforcement is primarily attributed to the combined reinforcing effects of rigid cellulose nanowhiskers and ceramic TiO_2_ nanoparticles. The intrinsic strength of CNWs and their effective dispersion within the PLA matrix [16,18] enable efficient load transfer from the polymer to the rigid nanofillers, resulting in enhanced stiffness. Furthermore, the synergistic effect between CNWs and Nano-TiO_2_ restricted the mobility of PLA molecular chains and acted as physical cross-linking points, further contributing to the modulus improvement [22]. In addition, the increased matrix crystallinity induced by heterogeneous nucleation (as confirmed by XRD) reduced the fraction of compliant amorphous regions and promoted tighter chain packing, which also increased the stiffness of the composite. Therefore, the synergistic effect of filler rigidity, interfacial stress transfer, and elevated crystallinity accounts for the notable enhancement in the tensile stiffness of the composite film. However, the elongation at break of the composite films experienced a sharp decline from 1.36% (PLA) to 0.02% (the composite film). This embrittlement is closely related to the formation of a rigid CNW-based network and the presence of numerous inorganic interfaces, which act as stress concentration points and suppress the plastic deformation capacity of the PLA matrix. The increased crystallinity (from 20.26% to 36.31%) further contributes to the enhanced modulus and strength, while simultaneously reducing the fraction of compliant amorphous regions responsible for ductility.

**Figure 6:**
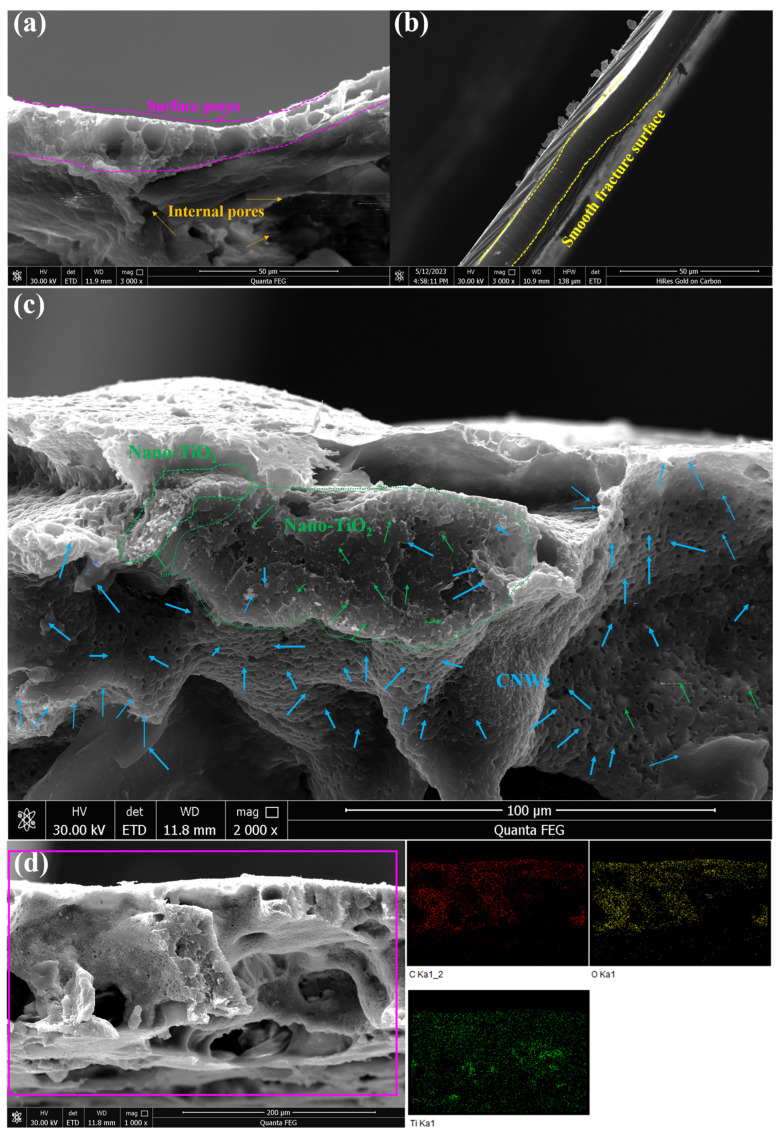
Cross-sectional SEM and EDS analysis of the films: (**a**) SEM image of the composite film exhibiting a hierarchical porous structure; (**b**) pristine PLA film; (**c**) high-magnification SEM image of the composite film, highlighting the embedded TiO_2_ nanoparticles (indicated by green arrows) and CNWs (indicated by blue arrows); (**d**) cross-sectional SEM image and the corresponding EDS elemental mapping of C, O, and Ti for the 1%CNWs-0.5%Nano-TiO_2_/PLA composite film.

The cross-sectional morphologies after the tensile test of the films were investigated using SEM. As illustrated in Figure 6b, the pristine PLA film exhibits a highly dense and continuous matrix with a distinctively smooth fracture surface. This morphology is characteristic of a homogeneous polymer film undergoing typical brittle or semi-brittle fracture. In contrast, the 1%CNWs-0.5%Nano-TiO_2_/PLA composite film induces a dramatic morphological transformation. As shown in Figure 6a, the composite film exhibits a markedly rough, highly porous, and hierarchical structure (Figure 6a,c). The cross-section reveals distinct “surface pores” distributed near the upper interface and interconnected “internal pores” extending throughout the whole composite film. In the magnified fracture surface in Figure 6c, numerous CNWs (indicated by blue arrows) and Nano-TiO_2_ particles (indicated by green arrows) can be observed. CNWs demonstrate significant pull-out phenomena and partial aggregation upon fracture. Meanwhile, TiO_2_ nanoparticles are firmly embedded within the polymeric matrix. During tensile loading, the well-dispersed Nano-TiO_2_ and CNWs act as effective stress-bearing centers, facilitating efficient stress transfer from the polymer matrix to the rigid CNWs and TiO_2_ nanoparticles. Furthermore, due to the pull-out process of CNWs, accompanied by interfacial friction and debonding, the CNWs effectively dissipate crack propagation energy, thereby leading to a remarkable improvement in the tensile strength of the composite film. Figure 6d is the corresponding EDS elemental mapping for the cross-section of the composite film, which illustrates the uniform distribution signals of C, O, and Ti elements. The successful incorporation and dispersion of CNWs and TiO_2_ nanoparticles within the composite matrix are further validated. The synergistic presence of these micro-voids, pulled-out CNWs, and distributed Nano-TiO_2_ reflects the complex interfacial interactions among the multiple components. As stated above, the significant enhancement in the tensile strength of the composite film is highly consistent with its microstructural features observed in the SEM cross-section.

**Figure 7:**
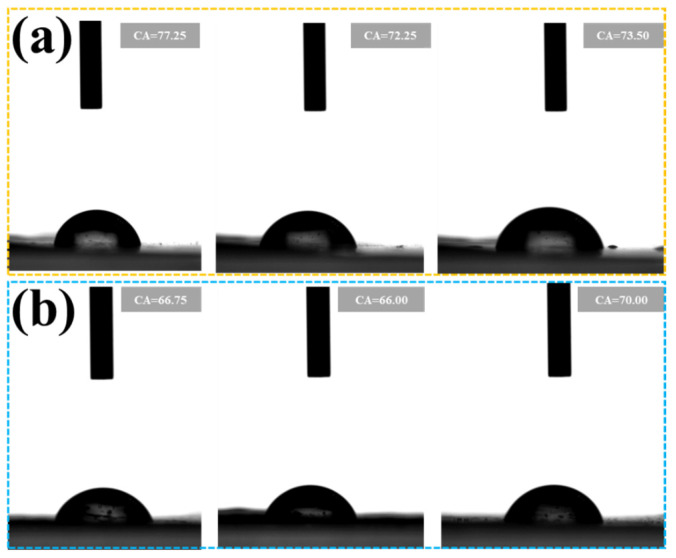
Water contact angle image of (**a**) pure PLA; (**b**) 1%CNWs-0.5%Nano-TiO_2_/PLA composite film.

The surface wettability of the composite film was evaluated using the water contact angle (WCA), as summarized in Figure 7 and Table 3. The water contact angle of the pure PLA film was 74.4 ± 2.6° (Figure 7a). After the incorporation of hydrophilic CNWs and TiO_2_ nanoparticles, the contact angle for the porous 1%CNWs-0.5%Nano-TiO_2_/PLA composites film decreased to 67.6 ± 2.1° (Figure 7b), indicating an improvement in surface wettability. This could be attributed to the presence of -OH functionalities from the CNWs and TiO_2_ molecules on the polymeric surface, thus conferring relatively high water-favoring characteristics [16,38]. In addition, the high hygroscopicity of CNWs gave it a strong moisture absorption capability [20], which is also beneficial for the reduced contact angle of the composite film.

### 3.3. Comprehensive Critical Comparison

To critically position the advancements of this work within the context of porous film developments, a comprehensive comparison with the recent literature on porous PLA-based materials is conducted and discussed in detail below.

Recent attempts to fabricate functional porous PLA films via the breath figure (BF) method or phase separation often grapple with a severe trade-off between mechanical endurance and functionality. For instance, Preuksarattanawut et al. [12] successfully prepared hierarchical honeycomb PLA films using calcium carbonate nanoparticles (CCNs) via a static breath figure method. While that work demonstrated tunable 2D/3D hierarchical porous architectures via CCN-assisted BF fabrication, the resulting films were primarily characterized for porosity, pore multilayering, and sensor application, with no quantitative reporting of tensile strength, Young’s modulus, or UV-shielding performance—key indicators of structural integrity and functional utility for packaging materials. Although porous PLA composites have been explored for environmental applications [39], such studies typically focus on sorptive/reactivity functionality without addressing the mechanical robustness or optical performance of the porous scaffold. Moreover, conventional BF-PLA films often suffer from compromised tensile strength post-poration. Other alternative modifications, such as chemical aminolysis or succinic anhydride grafting on porous PLA templates [40,41], inherently deteriorate the molecular weight or bulk continuity of the polymer chains during the template-leaching or chemical-etching processes, causing an unavoidable compromise in structural integrity.

In contrast, our strategy successfully breaks this conventional bottleneck through the co-incorporation of CNWs and Nano-TiO_2_. Instead of disrupting the breath figure formation, the well-dispersed Nano-TiO_2_ and rigid CNWs cooperatively assemble at the solvent–water droplet interface, operating as highly efficient Pickering stabilizers that prevent water droplet coalescence. Simultaneously, the highly crystalline CNWs interlock within the localized PLA matrix of the pore walls, forming a continuous, rigid nanoscale scaffolding network. This unique co-continuous reinforcement architecture not only ensures an exceptional functional performance (e.g., superhydrophobicity and self-cleaning) but also dramatically bolsters the film’s tensile properties and structural integrity.

### 3.4. Formation Mechanism of the Composite Film

**Figure 8:**
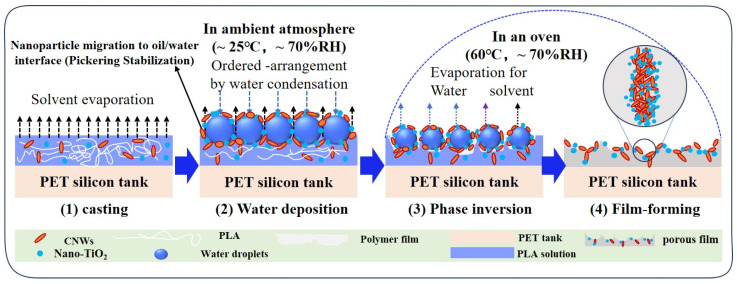
Schematic illustration of the formation mechanism of porous 1%CNWs-0.5%Nano-TiO_2_/PLA composite film stabilized by CNWs and TiO_2_ nanoparticles via the breath figure method.

The formation mechanism of the porous structure in the 1%CNWs-0.5% Nano-TiO_2_/PLA composite film via the breath figure method is illustrated in Figure 8. The process can be divided into four stages, demonstrating the critical role of nanoparticle-mediated Pickering stabilization.

Initially, the PLA solution containing well-dispersed CNWs (red rods) and Nano-TiO_2_ (blue dots) in dichloromethane is cast onto a PET silicon substrate at ambient conditions. Due to the rapid evaporation of the volatile dichloromethane solvent, the surface temperature of the casting solution drops significantly. Secondly, this temperature drop prompts the condensation of atmospheric water vapor (at ~70% relative humidity) into tiny water droplets on the solution surface. Driven by the reduction in interfacial system energy, both the CNWs and TiO_2_ nanoparticles spontaneously migrate from the bulk organic phase (PLA/DCM) towards the newly formed oil/water interface. As explicitly highlighted in the diagram, these nanoparticles accumulate and pack densely around the growing water droplets, forming a robust mechanical barrier. This is the characteristic of the Pickering stabilization effect. This particle armor effectively suppresses the coalescence of adjacent water droplets as they grow; consequently, the droplets were organized into a hexagonal close-packed array. Thirdly, the film is then transferred to an oven at 60 °C to promote phase inversion and solidification. As the continued evaporation of DCM increases the polymer concentration, the PLA matrix precipitates and solidifies around the stable, nanoparticle-encapsulated water droplets. The robust Pickering interface ensures that the ordered droplet array remains intact during this solidification process, preventing structural collapse. Finally, upon complete evaporation of the solvent and the water droplet templates, solid PLA film with a highly ordered, porous structure was obtained. Crucially, as depicted in the magnified inset circle, the CNWs and TiO_2_ nanoparticles are not randomly distributed throughout the bulk PLA matrix. Instead, they are likely to assemble on the inner walls of the pores (as shown in Figure 2c). This specific localization provides direct evidence of their adsorption at the oil/water interface during the early stages of the BF process, confirming that the highly ordered structure is attributed to the synergistic Pickering stabilization provided by the binary nanoparticle system.

## 4. Conclusions

In summary, a PLA composite porous film was prepared successfully through the breath figure method. The introduction of CNWs and Nano-TiO_2_ as Pickering-like stabilizers resulted in a uniform pore size distribution. The average pore sizes were about 2.49 ± 1.36 μm. The composite film exhibited better UV-shielding capabilities and mechanical properties than the pure PLA film, including a tensile strength of 30.1 MPa and a Young’s modulus of 0.386 GPa, but reduced ductility, which is attributed to the combined effects of filler reinforcement, interfacial stress transfer, and increased matrix crystallinity. Furthermore, the addition of hydrophilic nanoparticles improved the surface wettability of the films. Overall, these porous composite films with three-dimensional hierarchical structures combine the biodegradability of PLA with the functionality of nanoparticles, highlighting their potential for tissue engineering and functional packaging applications.

## Figures and Tables

**Table 1:** Quantitative optical properties of the PLA-based films.

Samples	Absorbance (at 300 nm)	SR*_UV-B_* (%)	Absorbance (at 360 nm)	SR*_UV-A_* (%)	T*vis* (%) (at 550 nm)
**Neat PLA**	0.05	10.9%	0.02	4.5%	94.2%
**1%CNWs-0.5%Nano-TiO_2_/PLA**	1.62	97.6%	0.88	86.8%	71.4%

**Table 2:** Tensile property data of the samples.

Samples	Tensile Strength (MPa)	Elongation at Break (%)	Young’s Modulus (GPa)	R^2^
PLA	13.48	1.36	0.32	0.998
	8.09	0.65	0.32	0.997
	1.33	0.65	0.32	0.996
1%CNWs-0.5%Nano-TiO_2_/PLA composite film	19.7	0.02	0.38	0.997
	21.2	0.02	0.39	0.996
	30.1	0.01	0.39	0.998

**Table 3:** Water contact angle of the films.

Composites Film	Water Contact Angle (°)			Average (°)
	1	2	3	
PLA	77.3	72.3	73.5	74.4 ± 2.6
1%CNWs-0.5%Nano-TiO_2_/PLA composite film	66.8	66	70	67.6 ± 2.1

## Data Availability

The original contributions presented in this study are included in the article. Further inquiries can be directed to the corresponding authors.

## Abbreviations

The following abbreviations are used in this manuscript:

CNWs	Cellulose nanowhiskers
Nano-TiO_2_	TiO_2_ nanoparticles
PLA	poly(lactic acid)

## References

[1] Cao T.T., Yabu H., Huh D.S. (2021). Flower-like ordered porous array by combination of breath figure and layer-by-layer technique. Polymer.

[2] Wang W., Feng M., Zou S., Chen C., Peng J., Li X., Zhang S., Ai X., Ma H. (2025). Strategies for fabrication and potential applications of conjugated microporous polymer films. Chin. Chem. Lett..

[3] Ali H., Orooji Y., Alzahrani A.Y.A., AL Mughram M.H., Abu-Dief A.M., Omar I., Hayat A., Yue D., Xu Y. (2025). Innovative morphological modifications of porous organic polymers for advanced photocatalytic applications. Coord. Chem. Rev..

[4] Zhao J., Zhu P., Zhang H. (2025). Preparation of Surface-Porous PS Films with a Submicron-Scale Pore Size from a PS/PEG–THF Ternary Mixture Based on Ultrasonic Dispersion. Langmuir.

[5] Moscolari L., Tullii G., Vignali A., Kozma E., Galeotti F. (2025). Combining Breath Figures with Mussel-Inspired Chemistry: An Easy Route to Finely Tunable Microporous Functional Surfaces. ACS Mater. Au.

[6] Aynard A., Pessoni L., Billon L. (2020). Directed self-assembly in “breath figure” templating of block copolymers followed by soft hydrolysis-condensation: One step towards synthetic bio-inspired silica diatoms exoskeleton. Polymer.

[7] Fajstavr D., Fajstavrová K., Frýdlová B., Slepičková Kasálková N., Švorčík V., Slepička P. (2023). Biopolymer Honeycomb Microstructures: A Review. Materials.

[8] Falak S., Shin B., Huh D. (2022). Modified Breath Figure Methods for the Pore-Selective Functionalization of Honeycomb-Patterned Porous Polymer Films. Nanomaterials.

[9] Kulshrestha P., Shin B.K., Prajapati K., Huh D.S. (2025). CdS and CdSe nanorod preparation by a bio-inspired process using breath figure method in the porous polymer film. Mater. Today Commun..

[10] Liu C., Wu M., Gao L., Liu H., Yu J. (2022). Nanoporous polymer films based on breath figure method for stretchable chemiresistive NO2 gas sensors. Sens. Actuat B-Chem..

[11] Boulett A., Marambio O., Martin-Trasanco R., Sánchez J., Alavia W., Oyarzún D.P., Pizarro G. (2023). Preparation of functional coating films using breath figure (BF) method and the study of morphological, optical and wettability behavior with varying experimental conditions. Int. J. Polym. Anal. Charact..

[12] Preuksarattanawut C., Kosolwattana S., Siralertmukul K., Ge F., Tsou C., Potiyaraj P., Nisaratanaporn E. (2024). Tunable honeycomb-hierarchical multiscale structures of 2D/3D porous PLA/CCN composite films fabricated by the breath figure method. Adv. Ind. Eng. Polym. Res..

[13] Huang J., Hao H., Huang Y., Yu B., Ren K., Jin Q., Ji J. (2021). Gradient Porous Structure Templated by Breath Figure Method. Langmuir.

[14] Chang M., Lee L., Huang M., Tsai T., Chen Y., Hong Y., Liu Y., Chen J. (2024). Light-Assisted Fabrication of Hierarchical Azopolymer Structures Using the Breath Figure Method and AAO Templates. Langmuir ACS J. Surf. Colloids.

[15] Wang H., Wang L., Liu C., Xu Y., Zhuang Y., Zhou Y., Gu S., Xu W., Yang H. (2019). Effect of temperature on the morphology of poly (lactic acid) porous membrane prepared via phase inversion induced by water droplets. Int. J. Biol. Macromol..

[16] Sucinda E.F., Abdul M.M., Ridzuan M., Cheng E.M., Alshahrani H.A., Mamat N. (2021). Development and characterisation of packaging film from Napier cellulose nanowhisker reinforced polylactic acid (PLA) bionanocomposites. Int. J. Biol. Macromol..

[17] Deghiche A., Haddaoui N., Zerriouh A., Fenni S.E., Cavallo D., Erto A., Benguerba Y. (2021). Effect of the stearic acid-modified TiO2 on PLA nanocomposites: Morphological and thermal properties at the microscopic scale. J. Environ. Chem. Eng..

[18] Chang X.X., Mubarak N.M., Karri R.R., Tan Y.H., Khalid M., Dehghani M.H., Tyagi I., Khan N.A. (2023). Insights into chitosan-based cellulose nanowhiskers reinforced nanocomposite material via deep eutectic solvent in green chemistry. Environ. Res..

[19] Neisi E., Dadkhah Tehrani A., Shamloei H.R. (2024). Development of cellulose nanowhisker–gallic acid antioxidant bioconjugate via covalent conjugation and supramolecular interactions: A comparative study. Int. J. Biol. Macromol..

[20] Al-Qahtani S.D., Al-Senani G.M. (2024). Green and sustainable smart wooden system integrated with cellulose nanowhiskers-supported polyvinyl alcohol and anthocyanin biomolecules to monitor food freshness. Spectrochim. Acta A.

[21] Khattab T.A., Fouda M.M.G., Rehan M., Okla M.K., Alamri S.A., Alaraidh I.A., AL-ghamdi A.A., Soufan W.H., Abdelsalam E.M., Allam A.A. (2020). Novel halochromic cellulose nanowhiskers from rice straw: Visual detection of urea. Carbohydr. Polym..

[22] Kian L.K., Jawaid M., Nasef M.M., Fouad H., Karim Z. (2021). Poly(lactic acid)/poly(butylene succinate) dual-layer membranes with cellulose nanowhisker for heavy metal ion separation. Int. J. Biol. Macromol..

[23] Sheng K., Zhang S., Qian S., Fontanillo Lopez C.A. (2019). High-toughness PLA/Bamboo cellulose nanowhiskers bionanocomposite strengthened with silylated ultrafine bamboo-char. Compos. Part B Eng..

[24] Lekesi L.P., Motaung T.E., Motloung S.V., Koao L.F., Malevu T.D. (2022). Investigation on structural, morphological, and optical studies of multiphase titanium dioxide nanoparticles. J. Mol. Struct..

[25] Shebi A., Lisa S. (2019). Evaluation of biocompatibility and bactericidal activity of hierarchically porous PLA-TiO2 nanocomposite films fabricated by breath-figure method. Mater. Chem. Phys..

[26] Shao L., Gong W., Wang C., Yin Y. (2025). Solution-blending strategy for multifunctional PLA/PCL/TiO_2_ composite films: Synergistic effects on mechanical reinforcement, ultraviolet shielding, and yellowing suppression. Polymer.

[27] Nayak J.K., Behera L., Jali B.R. (2024). TiO2 strengthened PLA nanocomposites: A prospective material for packaging application. J. Mol. Struct..

[28] Tajari N., Sadrnia H. (2025). Optimization of active PLA films using PROMETHEE method. Mater. Lett..

[29] Chanklom P., Kreetachat T., Chotigawin R., Suwannahong K. (2021). Photocatalytic Oxidation of PLA/TiO2-Composite Films for Indoor Air Purification. ACS Omega.

[30] Hong Q., Ma X., Li Z., Chen F., Zhang Q. (2016). Tuning the surface hydrophobicity of honeycomb porous films fabricated by star-shaped POSS-fluorinated acrylates polymer via breath-figure-templated self-assembly. Mater. Des..

[31] Zheng H., Li X., Liu L., Bai C., Liu B., Liao H., Yan M., Liu F., Han P., Zhang H. (2022). Preparation of nanofiber core-spun yarn based on cellulose nanowhiskers/quaternary ammonium salts nanocomposites for efficient and durable antibacterial textiles. Compos. Commun..

[32] Huang S., Zou S., Wang Y. (2023). Construction of compostable packaging with antibacterial property and improved performance using sprayed coatings of modified cellulose nanocrystals. Carbohydr. Polym..

[33] Shi J., Zhang L., Xiao P., Huang Y., Chen P., Wang X., Gu J., Zhang J., Chen T. (2018). Biodegradable PLA Nonwoven Fabric with Controllable Wettability for Efficient Water Purification and Photocatalysis Degradation. ACS Sustain. Chem. Eng..

[34] Widjaja T., Rohmah A.A.Z., Nurkhamidah S., Ni’Mah H., Wardhono E.Y., Saputra B.Y.E., Sari C.Y. (2025). Effectiveness study of Cellulose Nanocrystal (CNC) filler usage on polylactic acid (PLA) properties through plasticizer addition optimization: Application in paper-coated tableware. Case Stud. Chem. Environ. Eng..

[35] Kumar A., Dixit K., Sinha N. (2023). Fabrication and characterization of additively manufactured CNT-bioglass composite scaffolds coated with cellulose nanowhiskers for bone tissue engineering. Ceram. Int..

[36] Thakur M., Sharma A., Ahlawat V., Bhattacharya M., Goswami S. (2020). Process optimization for the production of cellulose nanocrystals from rice straw derived α-cellulose. Mater. Sci. Energy Technol..

[37] Cicogna F., Passaglia E., Benedettini M., Oberhauser W., Ishak R., Signori F., Coiai S. (2023). Rosmarinic and Glycyrrhetinic Acid-Modified Layered Double Hydroxides as Functional Additives for Poly(Lactic Acid)/Poly(Butylene Succinate) Blends. Molecules.

[38] Li S., Hu M., Chen X., Sui S., Jin L., Geng Y., Jiang J., Liu A. (2023). The performance and functionalization of modified cementitious materials via nano titanium-dioxide: A review. Case Stud. Constr. Mat..

[39] Špoljarić A., Bafti A., Vidović E. (2024). Synthesis of bare and modified zerovalent iron nanoparticles and their use in porous polylactic acid composites for methyl orange removal. J. Environ. Chem. Eng..

[40] Rafique A., Bulbul Y.E., Raza Z.A., Oksuz A.U. (2025). Free radical synthesis of succinic anhydride grafted poly(lactic acid) porous templates for sustained drug delivery in the buffer media. Int. J. Biol. Macromol..

[41] Rafique A., Bulbul Y.E., Raza Z.A., Oksuz A.U. (2024). Development of aminolyzed polylactic acid-based porous films for pH-responsive sustained drug delivery devices. Int. J. Biol. Macromol..

